# The Effect of Intravenous Magnesium Sulfate on Laryngospasm After Elective Adenotonsillectomy Surgery in Children

**DOI:** 10.5812/aapm.15960

**Published:** 2014-02-28

**Authors:** Shideh Marzban, Soudabeh Haddadi, Mohammad Reza Naghipour, Zahra Sayah Varg, Bahram Naderi Nabi

**Affiliations:** 1 Department of Anesthesia and Intensive Cares, Guilan University of Medical Sciences, Rasht, Iran; 2 Department of Epidemiology, Guilan University of Medical Sciences, Rasht, Iran

**Keywords:** Magnesium Sulfate, Laryngospasm, Surgical Procedures, Elective, Child

## Abstract

**Background:**

Laryngospasm is the protective reflex of tracheobronchial tree against secretions and hemorrhage. This reflex is more prevalent in adenotonsillectomy in the presence of light anesthesia, which can lead to obstruction of airway, complications, and mortality. Different methods have been studied for preventing this complication; however, none of them could reliably prevent it.

**Objectives:**

The objective was to assess the effect of magnesium sulfate on laryngospasm and coughing after adenotonsillectomy.

**Patients and Methods:**

Seventy children with three to 12 years of age and ASA classes I and II, who were candidates for adenotonsillectomy, were recruited in this randomized clinical trial. The study group received 15 mg/kg intravenous magnesium sulfate and the control group received 0.9% normal saline with the same volume, 2 minutes after tracheal intubation via intravenous infusion for 20 minutes. After removing the endotracheal tube in the recovery room, the patients were assessed at minutes zero, 15, and 30in terms of laryngospasm and coughing. The assessment was based on four-point scale of severity of these complications and saturation percentage of arterial oxygen in operating and recovery room. After collecting the data, results were analyzed with the SPSS 16 software anda P value < 0.05 was considered statistically significant.

**Results:**

Laryngospasm was not found in the magnesium sulfate group; however, its incidencewas5.7% in the control group. The incidence rates of coughs were 17.1% and 40% in the magnesium sulfate group and in the control group, respectively, which had no statistically significant differences.

**Conclusions:**

Intravenous magnesium sulfate with dose of 15 mg/kg could not prevent laryngospasm and coughing after removal of the endotracheal tube in patients undergoing adenotonsillectomy; however, it reduced coughing and laryngospasm in the magnesium sulfate group compared with the control group.

## 1. Background

Reflex laryngospasm or excessive closure of vocal cords, which is caused by stimulation of internal muscles of larynx, prevents entry of foreign bodies and surgical debridement into the tracheobronchial tree. Laryngospasm is more prevalent in surgeries of upper airways, particularly ear, nose, and throat surgeries such as tonsillectomy and adenotonsillectomy, which are accompanied by hemorrhage, secretions, and surgical debridement, especially in the presence of light anesthesia. This can lead to full obstruction of the upper airway after removing endotracheal tube (1, 2).

Annual incidence of laryngospasm is 17 per 1000 children younger than nine years old, which increases to 96 per 1000 children with upper respiratory tract infection (3). Incidence rate of laryngospasm after routine extubation in tonsillectomy varies between 12 and 25% (4). Incidence rate of laryngospasm is normally higher in children than adults, which is due to narrowness of larynx and smaller tracheal diameter in children that may be blocked by the edema after manipulation or trauma (5).

Laryngospasm plays a major role in unfavorable postoperative events. Death might issue as a result of either hypoxia and hypercarbia in primary stages or pulmonary edema due to negative pressure in delayed stages (4). These complications can lead to irreversible brain injuries; therefore, it is necessary to prevent and cure laryngospasm immediately. Different methods have been proposed for reducing laryngospasm, which include intravenous lidocaine (5), topical lidocaine (6), propofol (7), and etc.; however, none of them could reliably prevent laryngospasm. Post-extubation coughing and postoperative period may increase arterial blood pressure, heart rate, and intraocular and intracranial pressure and if severe enough, cause laryngospasm.

Magnesium sulfate is an important medication that suppresses central nervous system and can help increase depth of anesthesia. It also has calcium antagonist properties that increase flaccidity (8). In addition, magnesium has an inhibitory effect on smooth muscle contraction and may be useful in treatment of asthma (9). Amiralmomenin Hospital is the main center for ear, nose, and throat surgeries in Guilan Province and many adenotonsillectomy surgeries are performed on children in this center.

## 2. Objectives

Since laryngospasm is one of the main complications after extubation in this type of surgery and in this age group, and by considering theoretical effects of magnesium sulfate and complications following laryngospasm, the present work attempted to study the effect of magnesium sulfate on laryngospasm in children undergoing adenotonsillectomy and its usage in preventing laryngospasm or reducing its intensity.

## 3. Patients and Methods

After approval of the study protocol and its confirmation by the Vice Chancellor for Research of Faculty of Medicine, and recording the study in the website of clinical trials (IRCT201207181138N10), a double-blinded clinical trial was conducted on 70 children, three to 12 years of age with physical classes (ASA) I or II who were candidates for adenotonsillectomy. Patients with emergency surgeries, history of difficult intubation or extubation, history of cardiac, respiratory and renal diseases, supraglottitis, infections of upper respiratory system, atrioventricular block, history of myasthenia gravis, hypotension, record of passive smoking, repeated tonsillectomy due to hemorrhage of more than 100 ml during surgery, or surgery duration longer than 1.5 hour were excluded from the study. After providing the necessary information about the study and receiving the letter of consent from the parents of the children, the study was performed as follows: 

All children were evaluated one day before the surgery and were included in the study if they met the inclusion criteria. Using a computer, the patients were randomly assigned to one of the two groups receiving intravenous magnesium sulfate (study group) or water (control group).

The study was double-blinded. The patients and the anesthesia assistant (the person evaluating the effect of the medication) were not informed about the type of the prescribed drug. Only the anesthesiologist (the person prescribing the medication) was aware of the medications in order to take necessary measures in case of adverse medical complications.

When the patients were transferred to the operation room, intravenous lines were prepared and 5-10 cc/kg 0.9% normal saline serum was injected. After applying ECG, pulse oximetry, blood pressure monitoring, and precordial stethoscope placement, all the children were preoxygenated by 100% oxygen for 3 minutes.

Anesthesia was induced by injecting 0.02 mg/kg/iv atropine, 2 mic/kg/iv fentanyl, 3-5 mg/kg/iv thiopental sodium, and 1 mg/kg/iv lidocaine. Muscle relaxation was achieved using 0.15 mg/kg/iv cisatracurium. After controlling and governing the airway by an endotracheal tube, anesthesia maintenance was done using isoflurane 0-0.60% (inhalation anesthetic) and N_2_O 50% in oxygen. Immediately after intubation, 0.2mg/kg/iv dexamethasone was prescribed. In addition, 2 minutes after intubation, the patients of the study group received 15 mg/kg magnesium sulfate (Iran Hormone, Tehran, Iran) in 30 ml NaCl 0.9% (LLP, Tehran, Iran) for 20 minutes. The patients of the control group received water for injection (with similar volume to that of the Mg group) in 30 ml NaCl 0.9% (LLP, Tehran, Iran) during the 20 minutes. Magnesium sulfate is a colorless and odorless medication; because both magnesium sulfate and normal saline were prescribed to the patients based on their weight, they could not be differentiated at the injection time. In case of sensitivity, the medication injection was aborted and the patient was excluded from the study. During the surgery, ephedrine and 0.02 mg/kg/iv atropine were prescribed in case of hypotension (decreased BP of more than 20% of age-related BP) and bradycardia (decreased HR of more than 20% of age-related HR), respectively (10). All the patients were given 10 mg/kg acetaminophen suppository 20 minutes before the end of surgery. Finally, after return of spontaneous breathing, there was reversal of the muscle relaxant effect using 0.02 mg/kg/iv atropine and 0.04 mg/kg/iv neostigmine and the awake patients were extubated after suctioning the secretions. 

After extubation, the patients received oxygen and were transferred to the recovery room in tonsil position. ECG, pulse oximetry, and blood pressure monitoring was performed and 4-5 lit/min oxygen through a facemask was administered. Then, the children were assessed for the symptoms of laryngospasm, coughing, oxygen saturation, blood pressure, and heart rate until discharge from the recovery room. They were evaluated by a recovery nurse under direct supervision of the anesthesiologist from extubation time until transfer to the recovery room (T0) andat 15 (T1) and 30 minutes after staying in the recovery room. . In case the patients underwent surgery again, due to surgical complications such as severe hemorrhage from the surgery site, they were excluded from the study (high amount of blood causing laryngospasm).

If the volume of hemorrhage exceeded 100 cc (total blood available in the used suction and gauzes) during the surgery or the surgery lasted for longer than 1.5 hours, the patient was excluded from the study (excessive manipulation of the surgery site and severe hemorrhage during the surgery can cause laryngospasm and confounding factors).

Severity of laryngospasm was evaluated based on a four-point scale;0: lack of laryngospasm; 1: inhalation stridor; 2: complete obstruction of vocal cords; 3: cyanosis (5).

Coughing was also investigated using a four-point scale:0/ none , 1/ 1-3: slight coughing, 2/ 4-6: moderate coughing, 3/ above 6: severe or intermittent coughing (5).

Oxygen saturation was evaluated using pulse oximetry. SpO_2_of below 95% for 30 or more seconds was regarded as desaturation (5). After collecting data for analysis, SPSS software (v. 16) was used and statistical tests such as Chi-square, unpaired t-test, general linear model, and repeated measurement were used. In all tests, P value below 0.05 was considered statistically significant.

## 4. Results

Totally, 70 patients were included in the study (35 patients in magnesium sulfate and 35 patients in control groups). Generally, 38 participants (54.3%) were male and 32 (45.7%) were female. Mean age of the patients was 7.74 ± 1.66 years old; the youngest and oldest participants in both group shad five and 12 years of age, respectively. Participants of the two groups had no significant difference in terms of age and gender.

**Figure 1.**
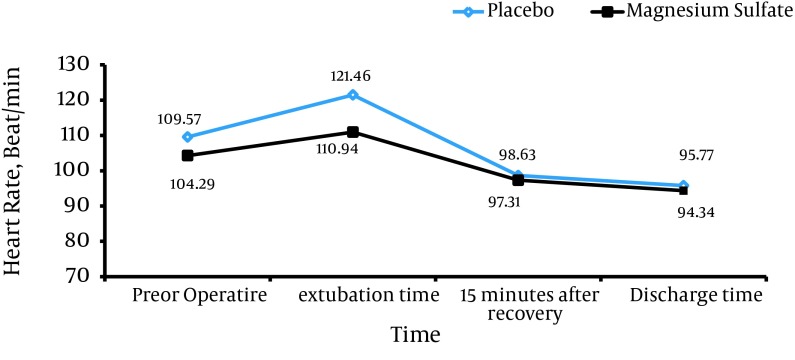
Changes in Heart Rate

To compare hemodynamic variables in both groups, trend of changes in terms of heart rate (beat/min) and mean arterial pressure (MAP) were compared at different intervals for both groups. Using general linear model and repeated measurement, it was specified that trend of changes in mean heart rate of the children did not show a statistically significant difference between the two groups and followed the same pattern (P = 0.097); however, changes in MAP in each group at different intervals were significantly different (P = 0.0001). Heart rate had a lower increase in magnesium sulfate while removing the endotracheal tube, this being statistically significant (P = 0.0001) (Figure 1). 

**Figure 2.**
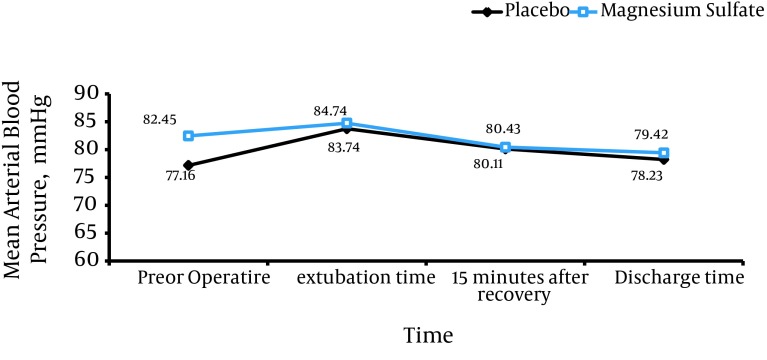
Changes in Blood Pressure

Using the general linear model and repeated measurement, it was specified that the trend of changes in MAP of the children at different intervals had no statistically significant difference between the two groups; in other words, changes in MAP at different intervals were not different and followed the same pattern (Figure 2). 

Laryngospasm was not found in the magnesium sulfate group; however, its incidence rate was 2 (5.7%) cases in the control group, which was not statistically significant. Both cases were found at the time of extubation, which had severity of one from the four-point scale. Inhalation stridor and no case of laryngospasm were observed in any other intervals (Table 1). 

**Table 1.** Incidence of Laryngospasm in the Two Groups, (n = 35) ^a^

Laryngospasm	MgSo_4_	Control	Total	P value
**+**	0	2 (5.7)	2 (2.9)	0.151
**-**	35 (100)	33 (94.3)	68 (97.1)	
**Total**	35 (100)	35 (100)	70 (100)	

^a^ Data are presented as No. (%).

In general, there was no statistically significant association between coughing incidence rate at different intervalsbetween the studied children in the two groups, those receiving magnesium sulfate (17.1%) and control (40%) (P > 0.05 in all, Table 2). Although severity of coughing was milder in the magnesium sulfate group, it was not statistically significant.

**Table 2.** Incidence of Cough in the Two Groups, (n = 35) ^a^

Cough	MgSo_4_	Control	Total	P value
**+**	6 (17.1)	14 (40)	20 (28.6)	0.063
**-**	29 (82.9)	21 (60)	50 (71.4)	
**Total**	35 (100)	35 (100)	70 (100)	

^a^ Data are presented as No. (%).

Mean O_2_ saturation percentage was compared between the two groups at different intervals. At thetime of discharge from the recovery room (30 minutes), this difference was statistically significant (P = 0.004). At other intervals, there was no statistically significant difference between the two groups in terms of mean O_2_ saturation (P > 0.05 in all, Table 3). 

**Table 3.** Comparing Mean O_2_Saturation Between the Two Groups

Time Interval	Mean ± SD	P value
**Extubation, (n = 35)**		0.181
MgSo_4_	98.11 ±1.25	
control	97.6 ±1.86	
**15 minutes after recovery, (n = 35)**		0.056
MgSo_4_	98.63 ±1.16	
control	98±1.51	
**30 minutes after recovery, (n = 35)**		0.004
MgSo_4_	99.37 ±0.84	
control	98.69 ±1.05	

## 5. Discussion

Airway management in children is one of the most important concerns of anesthesiologists. In upper airway surgeries like tonsillectomy, particularly among children younger than nine years of age, laryngospasm is more prevalent and can lead to obstruction of upper airway after removing endotracheal tube (1, 2). In this study, the effects of intravenous magnesium sulfate on laryngospasm after elective tonsillectomy in children were studied. Magnesium sulfate is also used for curing acute bronchospasm and severe asthma with dose of 10-25 mg/kg in children (11, 12).

Fuchs-Buder et al. reported that using 40 mg/kg magnesium sulfate did not cause considerable neuromuscular blockage and symptoms of muscular weakness in electromyography or clinic of the patient, and this dose was clinically safe (13). In this study, magnesium sulfate with dose of 15 mg/kg, which has been utilized in some studies on children, was used (8)

The incidence of laryngospasm after removing endotracheal tube in tonsillectomy varies between 12 and 25% and this rate increases in children due to their different airway anatomy from adults (4). Gulhas et al. studied 40 children undergoing tonsillectomy who randomly received 15 mg/kg intravenous magnesium sulfate after intubation and found no case of laryngospasm; however, incidence rate of laryngospasm in the placebo group was 25% (five out of 20 children) after removing endotracheal tube (8). In another study, Karaaslan et al. compared the effect of topical injection of bupivacaine-magnesium sulfate and bupivacaine alone on alleviation of pain and reduction of laryngospasm in 75 patients who underwent tonsillectomy. There was no significant difference between the two groups in terms of reduction of laryngospasm incidence (14). Batra et al. randomly injected propofol 60 seconds before extubation in elective adenotonsillectomy surgeries and found that laryngospasm incidence was 6.6% (four out of 60 children) in the group that received propofol and 20% (12 out of 60 children) in the placebo group (7). In our study, laryngospasm was not found in the magnesium sulfate group and laryngospasm incidence was 5.7% in the placebo group (two out of 35 children), which was not statistically significant. Results of this study were different from those by Gulhas. Although there was no significant difference between the two groups in terms of incidence of laryngospasm, in the present study, the absence of laryngospasm in magnesium sulfate was clinically valuable, particularly considering lower incidence of coughing compared with the control group, which indicated better bluntness of airway reflexes by magnesium sulfate.

Vahabi et al. compared 2 mg/kg topical magnesium sulfate 20% with normal saline in tonsillar fossa and found lower post-operative pain in the magnesium group, but the incidence of laryngospasm had no significant difference between the two groups (15). In contrast, laryngospasm incidence rate was lower in the magnesium sulfate group than the control group in our study.

In another study, Heidari et al. compared intravenous magnesium sulfate 15 mg/kg, lidocaine 1.5 mg/kg, and propofol 0.5 mg/kg in respiratory events after tonsillectomy. They concluded that respiratory events with these medications diminished, although none of them was superior to the others (16). In our study, laryngospasm incidence was lower in the magnesium sulfate group.

Different methods are used for preventing laryngospasm including accurate homeostasis during surgery to reduce hemorrhage, oropharyngeal suctioning only before removing endotracheal tube for movement of any remaining blood and secretions, and removing endotracheal tube in completely awake or deeply unconscious states. In this study, secretions and blood were removed by suctioning before removing endotracheal tube, which was removed in the fully awake state. 

Intravenous lidocaine is one of the medications that are used for treating and preventing laryngospasm due to suppression of airway reflexes with local anesthetics. In this study, 1 mg/kg intravenous lidocaine was used to reduce hemodynamic changes resulting from laryngoscopy during anesthesia induction. In addition, possible effect of lidocaine and dexamethasone on decreasing the incidence of laryngospasm and cough should be considered.

Hypoxemia is frequently caused after general anesthesia during the postoperative period (4). In a study conducted by Tsui et al. on children undergoing tonsillectomy by no-touch extubation technique, laryngospasm, oxygen saturation drop, and coughing were not found after removing the endotracheal tube (17). In the present study, the incidence of oxygen saturation drop in the control group was higher than the magnesium sulfate group (P < 0.0004) during discharge from the recovery room; however, such oxygen saturation drop did not drop below 95% in any of the groups. At other assessment points, oxygen saturation drop did not have statistically significant difference between the two groups. 

Post-extubation coughing and postoperative period increase arterial pressure, heart rate, and intraocular and intracranial pressure and cause laryngospasm in case of sever coughs. In the study by Ates et al. the incidence of laryngospasm and coughing among children who underwent ophthalmic surgery and had concurrent upper airway infection were 5% and 22%, respectively (18). However, in the present work, the incidence of coughing was 17.1% and 40% in magnesium sulfate and control groups, respectively. In fact, prescription of magnesium sulfate reduced the rate of coughing by approximately 33%, which was higher while removing endotracheal tube. Coughing severity in the control group was higher than in the magnesium sulfate group; however, this difference in severity was not statistically significant.

Intravenous magnesium sulfate with the dose of 15 mg/kg could not significantly prevent laryngospasm and coughing incidence after removing endotracheal tube among the patients undergoing tonsillectomy; however, it reduced laryngospasm and coughing incidence in magnesium sulfate group in contrast to the control group.

Oxygen saturation drop was lower in the magnesium sulfate group. This issue might be clinically important particularly in this age group with sensitivity to hypoxemia, which could be immediately afflicted with complications in case of negligence while removing endotracheal tube. In this study, magnesium sulfate reduced coughing incidence in comparison to the control group and coughing was more severe in the control group. Although difference in laryngospasm between the two groups was not statistically significant and by considering the cough incidence in the control group that was twice the rate of the magnesium sulfate group, it should be noted that significance of coughing is very high in case of large sample size. 

### 5.1. Suggestions

Considering the positive effect of magnesium sulfate on reduction of coughing and establishment of few intensive hemodynamic changes, it is recommended to use this medication in surgeries when post-extubation coughing can cause serious complications such as in neurosurgeries and ophthalmic surgeries. In further studies, effect of magnesium sulfate on coughing and neuromuscular block can is studied with larger sample sizes. In addition, it is recommended to use magnesium sulfate in children in upper airway surgeries to suppress airway reflexes and reduce coughing after removing endotracheal tube.

## References

[1] Henderson J, Miller RD (2010). Airway Management in the Adult.. Miller's Anesthesia..

[2] Mevorach DL (1996). The Management and Treatment of Recurrent Postoperative Laryngospasm.. Anesth Analg..

[3] Hampson-Evans D, Morgan P, Farrar M (2008). Pediatric laryngospasm.. Paediatr Anaesth..

[4] Allen M, Patel A, Miller RD (2010). Anesthesia for Eye,Ear,nose and throat surgery.. Miller’s anesthesia..

[5] Sanikop C, Bhat S (2010). Efficacy of intravenous lidocaine in prevention of post extubation laryngospasm in children undergoing cleft palate surgeries.. Indian J Anaesth..

[6] Staffel JG, Weissler MC, Tyler EP, Drake AF (1991). The prevention of postoperative stridor and laryngospasm with topical lidocaine.. Arch Otolaryngol Head Neck Surg..

[7] Batra YK, Ivanova M, Ali SS, Shamsah M, Al Qattan AR, Belani KG (2005). The efficacy of a subhypnotic dose of propofol in preventing laryngospasm following tonsillectomy and adenoidectomy in children.. Paediatr Anaesth..

[8] Gulhas N, Durmus M, Demirbilek S, Togal T, Ozturk E, Ersoy MO (2003). The use of magnesium to prevent laryngospasm after tonsillectomy and adenoidectomy: a preliminary study.. Paediatr Anaesth..

[9] Rowe BH, Bretzlaff JA, Bourdon C, Bota GW, Camargo CA, Jr. (2000). Intravenous magnesium sulfate treatment for acute asthma in the emergency department: a systematic review of the literature.. Ann Emerg Med..

[10] Zwass M, Gregory GA, Miller RD (2010). Pediatric and Neonatal Intensive Care.. Miller’s anesthesia..

[11] Leicht P, Wisborg T, Chraemmer-Jorgensen B (1985). Does intravenous lidocaine prevent laryngospasm after extubation in children?. Anesth Analg..

[12] Abou-Madi MN, Keszler H, Yacoub JM (1977). Cardiovascular reactions to laryngoscopy and tracheal intubation following small and large intravenous doses of lidocaine.. Can Anaesth Soc J..

[13] Fuchs-Buder T, Wilder-Smith OH, Borgeat A, Tassonyi E (1995). Interaction of magnesium sulphate with vecuronium-induced neuromuscular block.. Br J Anaesth..

[14] Karaaslan K, Yilmaz F, Gulcu N, Sarpkaya A, Colak C, Kocoglu H (2008). The effects of levobupivacaine versus levobupivacaine plus magnesium infiltration on postoperative analgesia and laryngospasm in pediatric tonsillectomy patients.. Int J Pediatr Otorhinolaryngol..

[15] Vahabi S, Shoja T, Chaibakhsh S, Khak M, Saljoughi N (2012). Effect of Postoperative Topical Administration of Magnesium Sulfate on Pain Relief in Paediatric Adenotonsillectomy: A Randomised Controlled Study.. HK J Paediatr..

[16] Heidari SM, Rahimi M, Hashemi SJ, Fesahat B (2013). A comparison between intravenous magnesium sulfate, lidocaine, propofol in prevention of respiratory complications after tonsillectomy.. J Isfahan Med Sch..

[17] Tsui BC, Wagner A, Cave D, Elliott C, El-Hakim H, Malherbe S (2004). The incidence of laryngospasm with a "no touch" extubation technique after tonsillectomy and adenoidectomy.. Anesth Analg..

[18] Ates Y, Alanoglu Z, Uysalel A (1998). Use of the laryngeal mask airway during ophthalmic surgery results in stable circulation and few complications: a prospective audit.. Acta Anaesthesiol Scand..

